# Traditional Chinese medicine for myasthenia gravis: Study protocol for a network meta-analysis

**DOI:** 10.1097/MD.0000000000021294

**Published:** 2020-07-17

**Authors:** Rongfang Xie, Liting Liu, Ruiqi Wang, Chunhua Huang

**Affiliations:** a Jiangxi University of Traditional Chinese Medicine; b Affiliated Hospital of Jiangxi University of Traditional Chinese Medicine, Nanchang, China.

**Keywords:** myasthenia gravis, network meta-analysis, protocol, traditional Chinese medicine

## Abstract

**Background:**

Myasthenia gravis (MG) is a disease that is difficult to cure, mainly manifested in the affected skeletal muscle weakness and morbid fatigue, which seriously affects the patients’ daily life and work. A large number of randomized controlled trial have shown that Traditional Chinese medicine (TCM) has a good effect in treating MG. However, due to the variety of TCM treatment methods, its relative effectiveness and safety have not been verified. Therefore, this study will use a network meta-analysis method to verify the effectiveness and safety of different types of TCM in the treatment of MG.

**Methods:**

We will search the following databases from inception to June 2020: the China National Knowledge Infrastructure, Wanfang Database, Chinese Science and Technology Periodical Database, Chinese Biomedical Literature Database, Pubmed, Embase, Web of Science, and the Cochrane library. Collect all randomized controlled trial of TCM for the treatment of MG, The 2 authors will independently select studies and extract data based on pre-designed inclusion and exclusion criteria. Methodological quality assessment and risk of bias will be assessed using Cochrane bias risk tool. All data analysis will be conducted using Revman5.3, WinBUGS 1.4.3, and Stata14.2 software.

**Results:**

This study will directly and indirectly compare the different outcome indicators of various studies, and rank the effectiveness of different TCM methods. The main outcome indicators include effectiveness, remission rate (no drug symptoms), relapse rate, clinical absolute score, and relative score. Secondary outcome indicators: including any related adverse reactions, the concentration of acetylcholine receptor antibody in serum.

**Conclusion:**

The conclusion of this systematic review will provide credible Evidence-based for the relative advantages of different TCM treatment methods for MG.

## 1 Introduction

Myasthenia gravis (MG) is an acquired autoimmune disease which mediated by acetylcholine receptor antibody, cellular immune dependence and complement participation and causes neuro-muscular junction transmission disorders.^[1,2,3,4]^ Its pathogenesis is complex, and it is not yet clear. Most scholars believe that its pathogenesis is related to genetics, immunity, and endocrine.^[5]^ Patients with MG mainly show fatigue of local or general skeletal muscle fatigue. Symptoms of drooping eyelids, weakness in swallowing, weakness in breathing, and general weakness are characterized by lighter in the morning and more severe at night, fluctuations during the day, and so on. The above symptoms will be relieved after rest, and activities will make the symptoms worse.^[6,7]^ Epidemiological surveys show that the prevalence rate of MG is between 10.66 and 32.89 per 100,000 people. Currently,^[8]^ the treatment of MG with Cholinesterase inhibitors, immunosuppressants, hormones, plasma exchange, intravenous gamma globulin, thymusectomy, and so on.^[9]^ These treatments can only relieve the symptoms of muscle weakness, delay the further development of the disease, and cannot cure the disease. And long-term use of them may cause serious adverse effects. For example, the symptoms of muscle weakness are temporarily exacerbated and the crisis of muscle weakness. Therefore, we need to further explore new treatment methods, traditional Chinese medicine (TCM) treatment may be a potential choice.

TCM is one of the world's overall medical systems. TCM has been used in China for thousands of years, and other countries in the world are also actively using it. TCM is already widespread and is considered to be promising to improve various diseases including MG. MG is classified as “Weizheng” in TCM. TCM believes that the cause and pathogenesis of MG are not single, so there are many corresponding treatment methods, such as acupuncture, Chinese herbal medicine, and so on. Acupuncture is based on meridian theory, which stimulates characteristic acupuncture points to clear meridians and smooth the body's qi and blood.^[10,11]^ Studies have shown that acupuncture treatment of MG will increase the expression of acetylcholine receptor at the neuromuscular junction.^[12]^ Studies have confirmed that the active ingredients of certain Chinese herbal medicines are flavonoid derivatives Can act as an acetylcholinesterase inhibitors, and so on. At present, TCM has been widely used for the treatment of MG due to its characteristics of low price, convenience, high efficacy, and few adverse reactions.^[13,14,15,16]^ Now there are many traditional meta-analysis that some TCM methods do have a good effect on the treatment of MG, However, all of these are direct comparisons between a single TCM and Western medicine, and there are no studies that directly or indirectly compare different TCM. As we all know, there are many kinds of TCM treatment methods, and the treatment advantages are different, which causes confusion to the choice of clinical operators.^[17,18]^ And network meta-analysis can make head-to-head comparisons of multiple interventions. Therefore, in order to solve the above-mentioned problems, we will use the network meta-analysis to systematically compare the effectiveness and safety of different TCM interventions, paving the way for future solutions for MG.

## 2 Methods

### 2.1 Protocol registration

The protocol has been registered on the INPLASY website (registration number is (INPLASY202060049). We will strictly abide by the requirements of the “the Preferred Reporting Items for Systematic Review and Meta-analysis Protocols” ^[19]^ to report the network meta-analysis. If there is any information adjustment during the entire study period, we will promptly correct and update it in the final report.

### 2.2 Inclusion and exclusion criteria

#### 2.2.1 Type of study

Only the study of randomized controlled trial (RCT) can be included, the language will be limited to Chinese or English. Exclude non-RCT, animal experiments, unclear results indicators such as images and other nonquantitative indicators. For the articles published repeatedly in Chinese and English journals, take the latest one published.

#### 2.2.2 Participants

Patients diagnosed with MG by internationally recognized diagnostic criteria, not restricted in age, gender, ethnicity, race, and disease stage. Reluctant to accept TCM treatment, patients with severe cardiovascular diseases, mental illnesses, and so on. will be excluded.

#### 2.2.3 Interventions

##### 2.2.3.1 Experimental interventions

The intervention measures of the experimental group were only TCM, such as Chinese herbal medicine, Chinese patent medicine, acupuncture, moxibustion, massage, and so on. It can be monotherapy or combination. RCT comparing the above 2 therapies can also be included, and those who combine Western medicine will be excluded.

##### 2.2.3.2 Control interventions.

The control group received conventional treatment of Western medicine, including the use of cholinesterase inhibitors, glucocorticoids, or a combination of both.

### 2.3 Outcome indicators

The main outcome indicators include effectiveness (recognized clinical efficacy evaluation criteria), effective including basic recovery, marked effect, improvement; remission rate (no drug symptoms), relapse rate,^[20]^ clinical absolute score, and relative score. Secondary outcome indicators: including any related adverse reactions, the concentration of acetylcholine receptor antibody in Serum.

### 2.4 Data sources and search strategies

We will search the following databases: the China National Knowledge Infrastructure, Wanfang Database, Chinese Science and Technology Periodical Database, Chinese Biomedical Literature Database, Pubmed, Embase, Web of Science, and the Cochrane library. Collect all the RCT on the treatment of MG with TCM. And manually search for references in related literature. The retrieval time is from the inception of the database to June 10, 2020. The language is limited to Chinese and English. The search strategy is to combine search terms with subject words and free words. The primary selection process are shown in PubMed search strategy (Table 1).

**Table 1:**
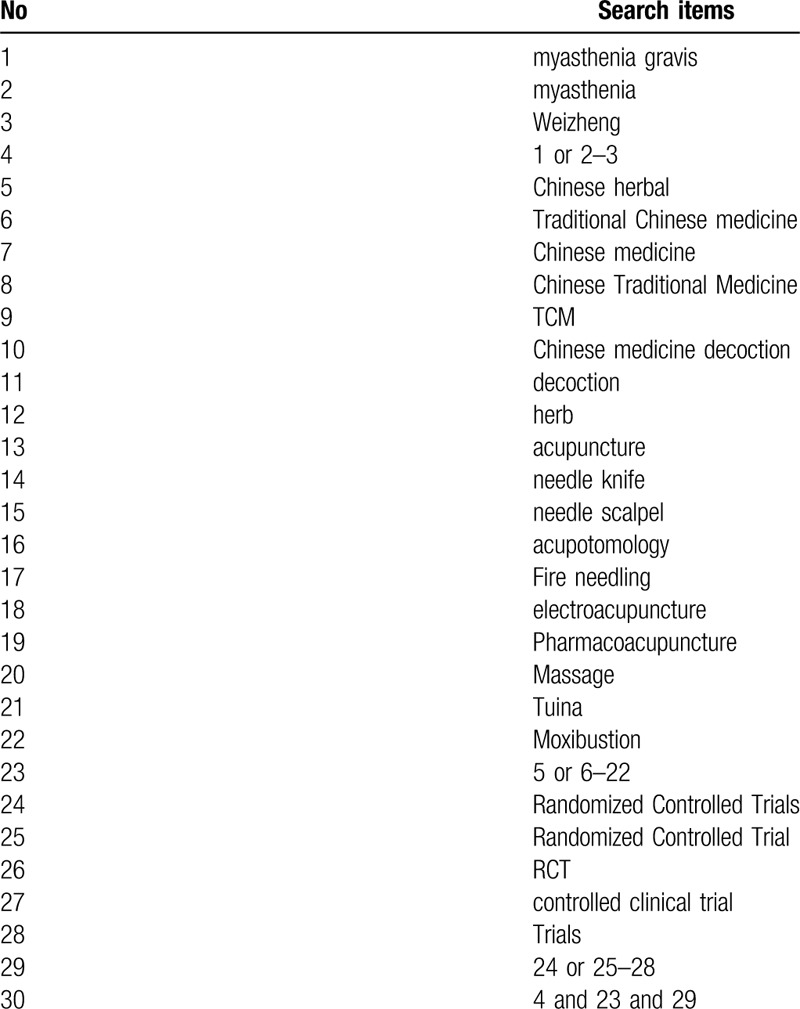
Search strategy used in PubMed database.

### 2.5 Selection of studies

**Figure 1:**
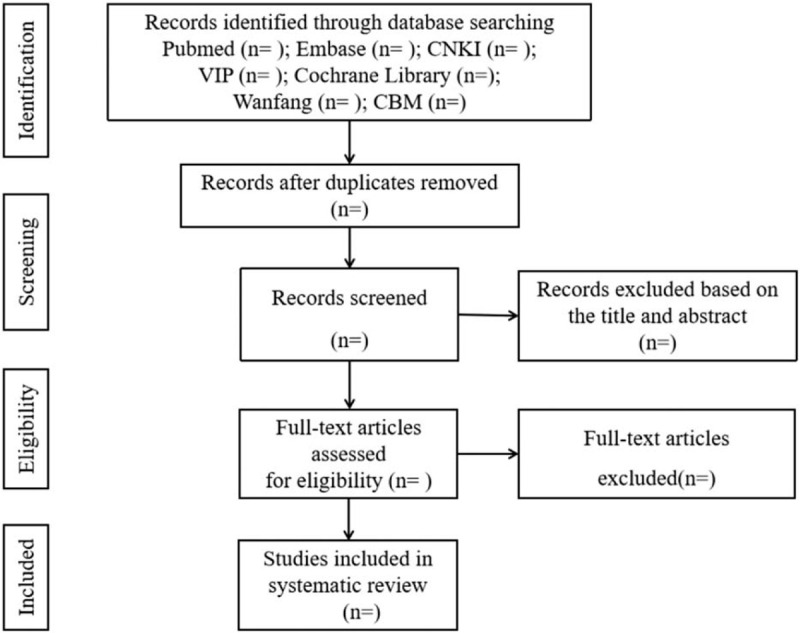
Flow diagram of study selection process.

Two authors independently complete the following process: according to the above search strategy to complete the process of document retrieval, import documents into EndNote X7 for centralized management. Then, according to the inclusion and exclusion criteria, filter the literature by reading the title and abstract. If it is not possible to determine whether the article meets the requirements based on the inclusion and exclusion criteria, then read the full text to select. In the entire literature screening process, if the 2 authors have different opinions, the third author joins the discussion to get a common opinion. The process of research selection is shown in Figure 1.

### 2.6 Data extraction

After the literature search process was completed, the 2 authors independently extracted the following information from the selected study: author, article title, year of publication, contact information, country/region, sample size, participants, diagnostic criteria, baseline characteristics, study design, random methods, blind methods, results, adverse events, and so on, and fill the extracted information into a pre-built Excel table. If necessary, we will contact the trial author for further information.

### 2.7 Dealing with missing data

If there is data loss in the included study, we will contact the original author of the article to obtain the original information. If the missing data is still not available, the existing data will be analyzed and a sensitivity analysis will be performed to address the potential impact of the missing data.

### 2.8 Risk of bias assessment and quality of selected studies

The 2 authors will independently assess the risk of bias (methodological quality) of the included studies based on the bias risk assessment tool recommended in the Cochrane “Risk of bias” assessment tool.^[21]^ Including 7 items: random sequence generation, allocation concealment, blind participants and personnel, blind assessment of results, incomplete result data, selective reports, and other biases. The results in each field will be divided into 3 levels: low bias risk, high bias risk, and unclear bias risk. The 2 authors will exchange assessment results and check whether the assessment results are consistent. If there is a disagreement, the third author will participate in the discussion and determine the final result.

### 2.9 Statistical analysis

Pairwise meta-analyses is conducted by RevMan5.3,^[22]^ Categorical data will be calculated with the risk ratio and 95% confidence intervals (CIs), continuous variables will be reported as mean differences or standardized mean differences with 95% CI. Heterogeneity will be evaluated by Chi-squared test and Higgins *I*^2^ test; If there is no obvious heterogeneity (*I*^*2*^ ≤ 50% and *P* > .10), the fixed effect model will be used; otherwise, the random effect model will be applied.

Use WinBUGS 1.4.3 and Stata14.2 for network meta-analysis. In WinBUGS 1.4.3 software, Bayesian framework is implemented by the Markov chain Monte Carlo method,^[23]^ which is simulated by 4 chains, the number of iterations is set to 50000, and the step size is set to 10. At the same time, the Potential Scale Reduced Factor (PSRF) is used to evaluate the convergence of the results. When the PRSF value is approximately equal to 1.00, it indicates that the results are well converged, and the obtained results are highly reliable. If the PRSF is not within this interval, then continue to manually increase the number of iterations 50,000 times until the FRSF is within this range. In the case of many interventions involved, in the evidence network of each outcome indicator, the closed loop formed by the research with direct and indirect evidence needs to be tested for inconsistency. Calculate the inconsistency factor (IF), and judge whether there is inconsistency by the IF value and the *P*-value. If the IF is close to 0, the 95% CI starts at 0, and *P* > .05, it is considered that the direct comparison and the indirect comparison are consistent.^[24]^ At the same time, the node-split model is used to determine whether each node has local inconsistency. If *P* > .05, there is no obvious inconsistency. If there is no obvious inconsistency between the 2, the consistency model is adopted, otherwise, the inconsistency model is adopted. For the results obtained by the analysis of the consistency model, the stability of the results can be checked by the inconsistency model.^[25,26]^

Make evidence network diagram, correct-compare funnel diagram, and conduct inconsistency test in Stata14.2 software. Simultaneously calculate the value of surface under the cumulative ranking curves and the area under the surface under the cumulative ranking curves curve in order to rank the efficacy of various interventions. The value range is 0 to 100. The larger the value, the larger the area under the curve indicates the intervention and the greater the likelihood of being the best intervention.

### 2.10 Subgroup analysis and sensitivity analysis

If the Chi-squared test and Higgins *I*^*2*^ test detect obvious heterogeneity between studies, we will conduct a subgroup analysis from the following aspects: different types of TCM, treatment time, MG classification, course of disease, and so on. In order to ensure the Credibility of the research results, we will conduct a sensitivity analysis of the included literature and will eliminate low-quality literature.

### 2.11 Publication bias

If the included studies are sufficient (n ≥ 10),^[27]^ the funnel plot will be used to assess the publication bias of the literature. If the funnel chart has poor symmetry, it indicates publication bias.

### 2.12 Assess the quality of evidence

The evaluation of the strength of the evidence will be based on the grading of recommendations assessment, development, and evaluation system, there are 4 levels of evidence strength: high, medium, low, or very low.

## 3 Discussion

In recent years, there have been more and more clinical reports of TCM treatment of MG, including a large number of RCT. There are also many traditional meta-analyses related to the treatment of MG with TCM, which shows that TCM has accumulated a profound theoretical basis and rich clinical experience in the treatment of MG. However, there are no reports of direct and indirect comparisons between different TCM interventions. Therefore, this study uses network meta-analysis to directly or indirectly compare different TCM interventions to find out which TCM interventions have relatively best efficacy and safety, and provide the best evidence for clinical practice.

## Author contributions

**Conceptualization:** Rongfang Xie.

**Methodology:** Rongfang Xie, Chunhua Huang.

**Project administration:** Ruiqi Wang

**Software:** Liting Liu

**Supervision:** Chunhua Huang.

**Writing – original draft:** Rongfang Xie.

**Writing – review & editing:** Chunhua Huang, Rongfang Xie.
